# Therapeutic Action of Fluoxetine is Associated with a Reduction in Prefrontal Cortical miR-1971 Expression Levels in a Mouse Model of Posttraumatic Stress Disorder

**DOI:** 10.3389/fpsyt.2013.00066

**Published:** 2013-07-10

**Authors:** Ulrike Schmidt, Leonie Herrmann, Kathrin Hagl, Bozidar Novak, Christine Huber, Florian Holsboer, Carsten T. Wotjak, Dominik R. Buell

**Affiliations:** ^1^RG Molecular Psychotraumatology, Max Planck Institute of Psychiatry, Munich, Germany; ^2^RG Neuronal Plasticity, Max Planck Institute of Psychiatry, Munich, Germany; ^3^Max Planck Institute of Psychiatry, Munich, Germany

**Keywords:** miRNA, miR-33, miR-1971, PTSD, PTSD mouse model, prefrontal cortex, SSRI

## Abstract

MicroRNAs (miRNA) are a class of small non-coding RNAs that have recently emerged as epigenetic modulators of gene expression in psychiatric diseases like schizophrenia and major depression. So far, miRNAs have neither been studied in patients suffering from posttraumatic stress disorder (PTSD) nor in PTSD animal models. Here, we present the first study exploring the connection between miRNAs and PTSD. Employing our previously established PTSD mouse model, we assessed miRNA profiles in prefrontal cortices (PFCs) dissected from either fluoxetine or control-treated wildtype C57BL/6N mice 74 days after their subjection to either a single traumatic electric footshock or mock-treatment. Fluoxetine is an antidepressant known to be effective both in PTSD patients and in mice suffering from a PTSD-like syndrome. Screening for differences in the relative expression levels of all potential miRNA target sequences of miRBase 18.0 by pairwise comparison of the PFC miRNA profiles of the four mouse groups mentioned resulted in identification of five miRNA candidate molecules. Validation of these miRNA candidates by reverse transcriptase quantitative polymerase chain reaction (RT-qPCR) revealed that the therapeutic action of fluoxetine in shocked mice is associated with a significant reduction in mmu-miR-1971 expression. Furthermore, our findings suggest that traumatic stress and fluoxetine interact to cause distinct alterations in the mouse PFC miRNA signature in the long-term.

## Introduction

Posttraumatic stress disorder (PTSD) is a debilitating anxiety disease occurring in about 2–9% of individuals after their exposure to life-threatening events like severe accidents, sexual abuse, combat, or natural catastrophes (1, 2). Although selective serotonin reuptake inhibitor (SSRIs) antidepressants like fluoxetine are currently the first line choice in PTSD drug treatment (3, 4), the response rates to SSRI treatment rarely exceed 60% and less than 20–30% of SSRI-treated PTSD patients achieve full remission (5). This unsatisfactory situation together with the fact that there is currently no drug available that specifically tackles PTSD core symptoms (3, 5), namely re-experiencing of traumatic memories, nervous hyperarousal, and avoidance of trauma-related cues (6) stresses the urgent need for development of novel PTSD-specific drugs and hence for elucidation of the, as yet insufficiently explored, molecular basis of PTSD. Epigenetic mechanisms increasingly emerge to play a role in PTSD pathobiology (7), i.e., it was recently discovered that allele-specific DNA demethylation of *FKBP5*, a potential PTSD biomarker (8), mediates gene × childhood trauma interactions (9). Furthermore, epigenetic regulation of immune-system associated molecules (10) and of catechol-*O*-methyltransferase (COMT), an enzyme which is critical for regulation of synaptic dopamine, was reported to be altered in PTSD patients (11). Besides DNA methylation and histone modifications, some authors consider small non-coding RNAs, like the about 22 nucleotides (nt) long miRNAs, as epigenetic regulators (12, 13). However, the view of miRNAs as regulators of epigenetic processes as well as reports on the epigenetic regulation of miRNA expression are more common (14, 15). miRNAs are well conserved in eukaryotic organisms (16) and play a pivotal role in regulation of posttranscriptional gene expression (12). They are encoded by eukaryotic DNA and function via base-pairing with complementary sequences of mRNA molecules through rapid mRNA decay and direct translational repression (17). MiRNAs have been associated not only with cancer (14) and autoimmune diseases (18) but also with psychiatric disorders like schizophrenia, autism (19), major depression (20), and anxiety diseases like panic disorder and specific phobias (21). In mice, expression of miR-128b was found to be increased in infralimbic prefrontal cortices (PFCs) in response to fear extinction training (22) which is considered to model exposure-based therapy (23), a psychotherapeutic strategy applied *inter alia* in PTSD patients (24). Furthermore, there is much evidence for miRNAs to play an important role in relation to the epigenetic tuning of the stress response (25, 26). For example, stress was shown to up-regulate mi34c expression in mouse amygdala and, moreover, lentivirally overexpressed mi34c was reported to induce anxiolytic-like behavior after challenge (27). Interestingly, to the best of our knowledge, miRNA regulation, expression, and function have so far not been studied at all in PTSD, neither in PTSD patients nor in PTSD animal models. Here, we present the first study exploring the connection between miRNAs and the PTSD-like syndrome in rodents. Using a miRNA microarray, we analyzed miRNA profiles in our previously established mouse model for PTSD (28, 42). In detail, we compared miRBase 18.0 based miRNA profiles in PFC samples of four groups of mice, i.e., footshocked and non-footshocked mice which were either fluoxetine-treated or untreated. We chose the PFC for miRNA profile analysis since this brain region was found to be reduced in volume (30) as well as altered in function (31, 32) in PTSD patients. Moreover, since in the PTSD model studied here we found shocked mice to exhibit an increased conditioned fear response, the notion that the PFC, beyond its known function in fear extinction (33), increasingly emerges to play a role in fear conditioning (33, 34) further sparked our interest in this brain region. In addition, prefrontal cortical miRNA expression levels have been reported to be altered in other psychiatric disorders: for instance, let-7d was shown to be up-regulated in the PFC of spontaneous hyperactive rats, an animal model for attention deficit hyperactivity disorder (ADHD) (35), and miR-195 was demonstrated to fine-tune regional levels of brain derived neurotrophic factor (BDNF) in the PFC of schizophrenic patients (36).

## Materials and Methods

### Animals

All experimental procedures were approved by the Committee on Animal Health and Care of Upper Bavaria (Regierung von Oberbayern), Germany (approval ID-AZ: 55.2-1-54-2531-41-09) and were conducted according to the current regulations for animal experimentation in Germany and the European Union (European Communities Council Directive 86/609/EEC). Twenty-three days old male C57BL/6NCrl mice purchased from Charles River GmbH (Sulzfeld, Germany) were housed in groups in the animal facility of the Max Planck Institute (MPI-P) for 6 weeks under an inverse 12:12 h light-dark cycle (lights off: 09:00 a.m.) with food and water *ad libitum*.

### PTSD mouse model

Experiments were performed during the activity phase of the mice, i.e., between 9:30 a.m. and 6:00 p.m., employing our established PTSD mouse model which we described in detail previously (28, 29). Briefly, 10-week-old male C57BL/6NCrl mice were subjected to a single 1.5 mA electric footshock for 2 s or mock treatment (exposure to shock chamber, the latter is termed “shock context” or “shock chamber” in the following). Beginning the day after footshock or mock treatment, half of the footshocked and half of the mock-treated mice received oral fluoxetine treatment (*n* = 6 per group). Thus, we compared four groups of mice, i.e., footshocked and mock-treated mice, which were either fluoxetine (Ratiopharm, Ulm, Germany) or vehicle-treated; these groups are termed “no-shock-vehicle,” “no-shock-fluoxetine,” “shock-vehicle,” and “shock-fluoxetine” in the following. Fluoxetine was administered in drinking water in a dose of 20 mg/kg/day for 28 days. The control group received drinking water only. On day 28 after footshock or mock treatment, fluoxetine efficacy was assessed by evaluation of their generalized fear response for 60 s during the presentation of a neutral tone (80 dB, 9 kHz) in a neutral context. Subsequently, the dose of fluoxetine was halved (10 mg/kg/day) and treatment was further continued for 3 days until discontinuation on day 31. Then, 59–60 days after footshock or mock treatment, hyperarousal was assessed by evaluation of their acoustic startle response. In addition, their generalized fear response was analyzed by monitoring their freezing behavior upon subsequent exposure to a neutral experimental context and to an experimental context similar to the shock chamber. Finally, the conditioned fear response of the mice was assessed by measuring their freezing behavior during (re-)exposure to the shock chamber. Video-taped animal behavior was rated off-line by a trained observer who was blind to the experimental conditions. Statistical analysis of behavioral data was performed using two-way ANOVA and Bonferroni *post hoc* tests.

### RNA extraction

Seventy four days after footshock or mock treatment, mice were sacrificed by cervical dislocation and PFCs were dissected (*n* = 6 per group). Total RNA was extracted employing the TRIzol^®^ protocol following the manufacturer’s instructions (Invitrogen, Paisley, UK). Extracted total RNA was resolved in nuclease free water. Concentrations of total RNA were assessed spectrophotometrically with a Nanophotometer (Implen GmbH, Munich, Germany). RNA integrity was assured by Agilent 2100 Bioanalyzer (Agilent Technologies, Inc., Santa Clara, CA, USA) both in our laboratory and in the laboratory of the microarray service provider (Exiqon A/S, Vedbaek, Denmark). RNA integrity numbers were ≥8.90 throughout the samples and all samples exhibited clear 18S and 28S RNA peaks in Bioanalyzer profiles.

### miRCURY LNA™ miRNA microarray profiling

RNA samples (6 × 4 = 24 samples) were shipped from the MPI-P in Munich to the microarray service provider Exiqon (Exiqon A/S, Vedbaek, Denmark) where all miRNA microarray experiments were performed. Accordingly, the chapter at hand (description of miRNA microarray procedure) is based on information provided by Exiqon (Exiqon A/S, Vedbaek, Denmark): 600 ng of total RNA extracted from samples were labeled with fluorescent Hy3™ and 600 ng of total RNA from reference probe with fluorescent Hy5™ using the miRCURY LNA™ miRNA Hi-Power Labeling Kit (Exiqon A/S, Vedbaek, Denmark) according to the manufacturer’s protocol. The Hy3™ -labeled samples were mixed pairwise with a Hy5™ -labeled reference probe and hybridized to the miRCURY LNA™ miRNA Array 7th Gen (Exiqon A/S, Vedbaek, Denmark) which contains capture probes targeting all miRNAs registered in the miRBase 18.0 (human, mouse, or rat)^1^ as well as viral miRNAs related to these species. Hybridization was performed according to the miRCURY LNA™ miRNA Array Instruction manual (Exiqon A/S, Vedbaek, Denmark) using a Tecan HS4800™ hybridization station (Tecan Austria GmbH, Salzburg, Austria). After hybridization, microarray slides were scanned and stored in an ozone free environment (ozone below 2.0 ppb) in order to prevent potential bleaching of fluorescent labels. The miRCURY LNA™ miRNA Array slides were scanned using the Agilent G2565BA Microarray Scanner System (Agilent Technologies, Inc., USA). Image analysis was carried out with ImaGene^®^ 9 miRCURY LNA™ miRNA Array Analysis Software (Exiqon A/S, Vedbaek, Denmark).

### Microarray data processing

Pre-processed microarray data was provided by Exiqon (Exiqon A/S, Vedbaek, Denmark). Accordingly, the description of microarray data processing is based on information provided by Exiqon: Signal intensity was the basis of data filtering. Background correction of quantified signals was performed via subtraction of the median global background from the median local background from the intensity of signals (Normexp with offset value 10) and resulted in the exclusion of two samples of the experimental group “no-shock-fluoxetine” (Figures 3B and 4). Normalization of data was performed with the global Lowess (locally weighted scatterplot smoothing) regression algorithm (37). All calculations have been performed using the software R/bioconductor employing mainly the limma package. Comparisons of miRNA expression values between experimental groups were performed using moderated *t*-statistics with standard errors moderated across genes, i.e., shrunk toward a common value, using a simple Bayesian model. This has the effect of borrowing information from the ensemble of genes to aid with inference about each individual gene (38). *P*-values were corrected for multiple testing by the Benjamini and Hochberg adjustment method to control for false positive results.

With the corrected *p*-values delivered by Exiqon (Exiqon A/S, Vedbaek, Denmark), we performed unsupervised hierarchical clustering analyses (HCA) in which we included the top 50 miRNA candidates with the lowest corrected *p*-values. HCA results are depicted in heatmaps which we generated by a web-based tool provided by the Los Alamos National Laboratory HIV sequence database^2^. For HCA, the complete-linkage method together with the Euclidean distance measure was employed. Complete-linkage clustering (by Euclidean distance) between sample subsets is represented by dendrograms (Figures 2–4).

### Reverse transcriptase quantitative PCR

For reverse transcriptase quantitative polymerase chain reaction (RT-qPCR), which was performed at the MPI-P, we employed either pre-designed LNA™ PCR primer sets for miRCURY LNA™ Universal RT microRNA PCR or Custom LNA™ PCR primers (UniRT) (Exiqon A/S, Vedbaek, Denmark). A list of all primer sets and their respective target sequences used is provided in Table 1. We used the miRCURY LNA™ miRNA PCR system first strand synthesis kit for poly-adenylation (poly-A-tailing) and reverse transcription (input of total RNA: 100 ng) according to the manufacturer’s protocol (Exiqon A/S, Vedbaek, Denmark). Then, 1 μl of 1:80 diluted cDNA was amplified by RT-qPCR in 5 μl SYBR Green PCR master mix containing 0.25 mM of LNA™ miRNA specific primer sets (Table 1). The total reaction volume was 10 μl. RT-qPCR was performed on the LightCycler^®^ 480 instrument (Roche Diagnostics, Penzberg, Germany). Each sample was analyzed in duplicate in every run, i.e., for each miRNA candidate tested. Cycling conditions were as follows: denaturation step 95°C 5 min followed by 45 loops of a two-segment amplification step (95°C, 30 s, 62°C, 1 min). A standard curve was generated for each individual plate assay with 1:10, 1:100, and 1:1000 dilutions and PCR efficiencies were calculated. *C*_p_ values were obtained with the software provided by the manufacturer (Roche Diagnostics, Penzberg, Germany). MiRNA entities for normalization were selected via NormFinder analysis based on microarray data (39) and mmu-miR-100-5p was used for normalization. Relative expression was calculated by the ΔΔ*C*_t_ method (40).

**Table 1:** **List of primer sets used for RT-qPCR**.

Target name	Product no./design ID (custom)	Target miRNA sequence
mmu-miR-33-5p	204632	GUGCAUUGUAGUUGCAUUGCA
mmu-miR-100-5p	204133	AACCCGUAGAUCCGAACUUGUG
mmu-miR-1971	206999 (custom)/design ID 212160	GUAAAGGCUGGGCUGAGA
mmu-miR-1947-3p	206999 (custom)/design ID 212154	GCACUGAGCUAGCUCUCCCUCC
rno-miR-3559-3p	206999 (custom)/design ID 212147	AUGUAGUACUGAGUCUGUCGUG
ebv-miR-BART8-3p	206999 (custom)/design ID 212150	GUCACAAUCUAUGGGGUCGUAGA

*We employed either pre-designed LNA™ PCR primer sets for miRCURY LNA™ Universal RT microRNA PCR or Custom LNA™ PCR primers (UniRT) (Exiqon A/S, Vedbaek, Denmark). The primer sets are designed for detection of the respective target sequences*.

### miRNA target prediction and gene ontology analysis

Materials and methods for miRNA target prediction and gene ontology (GO) analysis are described in detail in the Section “Results.”

## Results

### Fluoxetine counteracts the long-lasting PTSD-like syndrome in mice

**Figure 1:**
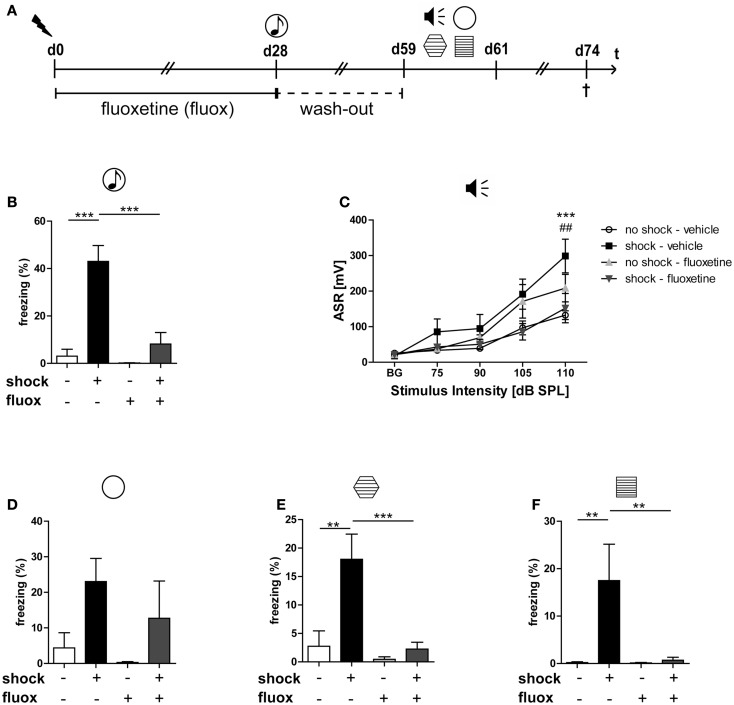
**Fluoxetine counteracts the long-lasting PTSD-like syndrome in mice**. Male C57BL/6NCrl mice (*n* = 6 per group) were either subjected to a single electric footshock 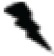 (“shock”) or, mock treatment (“no-shock”). Subsequently, both shocked and non-shocked mice were treated with either fluoxetine (20 mg/kg/day) (“fluoxetine”) or, for control, with drinking water (“vehicle”) for 28 days **(A)**. On day 28 after footshock or mock-treatment their freezing response to a neutral tone was assessed in a neutral experimental context 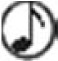 (generalized fear response) **(B)**. On day 29, the dose of fluoxetine was halved (i.e., 10 mg/kg/day) prior to treatment discontinuation on day 31. On days 59–61, PTSD-like behavior was analyzed: first, we assessed the intensity of the acoustic startle reflex (ASR) 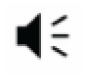
in response to white noise pulses of 50 dB (background, BG) and 75, 90, 105, and 115 dB **(C)**. Then, we evaluated the generalized fear response by assessment of the freezing response both in a neutral experimental context 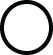 **(D)** and in a grid context similar to the shock chamber 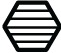 **(E)**. Finally, the conditioned fear response was analyzed by evaluation of the freezing response in the shock context (re-exposure to shock chamber) 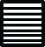 **(F)**. Freezing duration was assessed for a total of 3 min. The absolute time of immobility except respiratory movements was normalized to this 3 min observation interval (Freezing [%]). Presented data are means ± SEM. Statistical analysis was performed using two-way ANOVA and Bonferroni *post hoc* tests. Statistical significance of Bonferroni *post hoc* tests is indicated, for comparison of the groups “no-shock-vehicle” versus “shock-vehicle” by *p < 0.05, **p < 0.01, ***p < 0.001; respectively; comparison of groups “shock-vehicle” versus “shock-fluoxetine” by ^##^*p* < 0.01. See Section “Results” for statistical data.

To analyze the impact of traumatic stress on miRNA profiles in mouse PFC, we employed a well-established mouse model for PTSD that we (28, 29, 41–43) and also other research groups used, at least in slightly modified ways (44–46), for previous experiments. The electric footshock-elicited murine PTSD-like syndrome can be effectively counteracted by the orally administered SSRI antidepressant fluoxetine (28, 29) and, as we published recently, lasts at least until day 60 after shock application (28). First, we had to re-establish the behavioral syndrome-inducing effect of footshock and the relieving action of fluoxetine in the mouse cohort studied here: in contrast to our expectations (28, 29), and despite a significant effect of shock (*F*_1,20_ shock = 5.696, *p* = 0.027), the relative increase of the generalized fear response of shocked mice in the *neutral* context was not statistically significant after Bonferroni correction on day 60 (Figure 1D) but at least on day 28 (Figure 1B, *t* = 6.461, *p* < 0.001). The results of the other behavioral experiments turned out as expected: on day 59/60 after their subjection to shock, in comparison to mock-treated control mice, shocked mice exhibited a significantly stronger generalized fear response (in a context similar to the shock chamber) (Figure 1E, *t* = 4.058, *p* < 0.01) as well as more pronounced acoustic startle (Figure 1C, *t* = 4.468, *p* < 0.001) and conditioned fear responses (Figure 1F, *t* = 3.609, *p* < 0.01). Moreover, fluoxetine treatment drastically reduced trauma-mediated behavioral changes (Figure 1B: *t* = 5.630, *p* < 0.001, Figure 1C: *t* = 3.939, *p* < 0.01, Figure 1E: *t* = 4.193, *p* < 0.001, Figure 1F: *t* = 3.505, *p* < 0.01).

The behavioral consequences of stress exposure make this mouse model an animal model of PTSD, not the type or intensity of the stressor applied. The relatively increased conditioned and generalized fear responses in footshocked mice mirror the PTSD-associated avoidance behavior in humans: In most PTSD patients, the aversive avoidance of trauma-related reminders generalizes over time in sense that someday also trauma-unrelated cues suffice to elicit an intense avoidance anxiety. Moreover, the relatively increased startle response in footshocked mice has been repeatedly described also in PTSD patients (47–49). Hence, it reflects trauma-elicited nervous hyperexcitability in both men and mice. Other PTSD animal models employ more intense stressors in order to better model their traumatizing nature (50, 51).

### Traumatic footshock *per se* does not significantly alter mouse PFC miRNA profiles in the long-term

**Figure 2:**
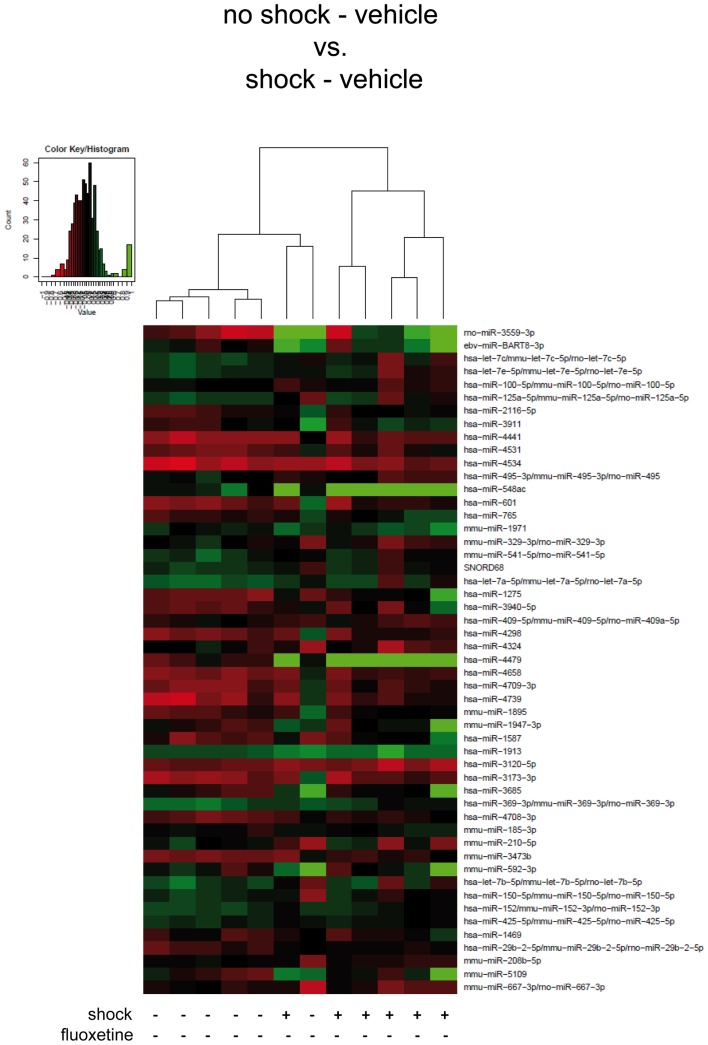
**Traumatic footshock does not significantly alter mouse PFC miRNA profiles in the long-term**. Here, results of the hierarchical cluster analysis (HCA) of the footshocked vehicle-treated (shock-vehicle) versus the non-shocked vehicle-treated (no-shock-vehicle) samples are presented in a heatmap. The top 50 miRNA candidates with the lowest corrected *p*-values (resulting from pairwise comparison of the two groups shown here) were included. MiRNA expression levels were determined with LNA™ miRNA microarray analysis of mouse prefrontal cortex (PFC) total RNA samples. Samples were collected on day 74 after footshock (“shock”) or mock treatment (“no-shock”) from male C57/BL6/N mice (*n* = 6 per group). Each row represents a miRNA and each column represents a sample. Dendrograms represent complete-linkage clustering (by Euclidean distance) between samples. The sample clustering tree is shown on the top. The color scale illustrates the intensities of the relative miRNA expression levels: decreased scores are represented in red and increased in green, with intensity encoding magnitude. See Section “Materials and Methods” for statistical procedures. Vehicle, drinking water (solvent of fluoxetine).

**Figure 3:**
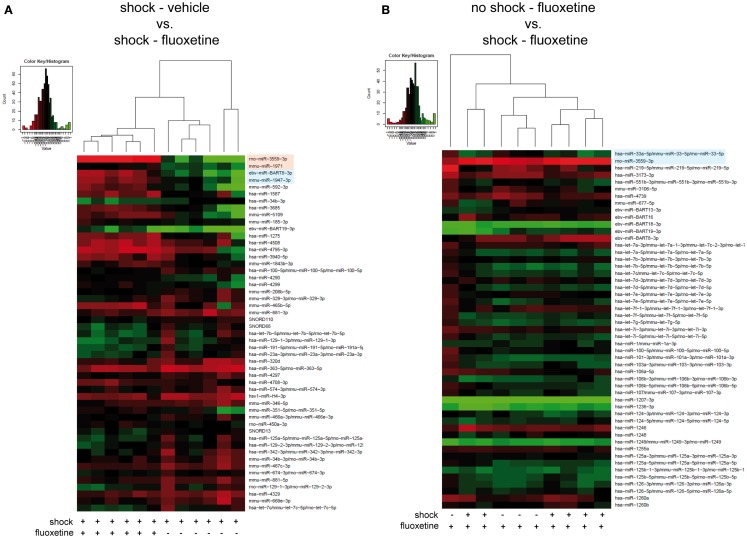
**Microarray analysis revealed fluoxetine treatment to significantly alter the expression of several miRNAs in the PFC of shocked mice**. Here, results of the hierarchical cluster analysis (HCA) of **(A)** the footshocked vehicle-treated (shock-vehicle) versus the footshocked fluoxetine-treated (shock-fluoxetine) groups and of **(B)** the non-shocked fluoxetine-treated (no-shock-fluoxetine) versus the footshocked fluoxetine-treated (shock-fluoxetine) groups are presented in heatmaps. The top 50 miRNA candidates with the lowest corrected *p*-values [resulting from pairwise comparison of groups shown in **(A,B)**, respectively] were included. MiRNA expression levels were determined with LNA™ miRNA microarray analysis of mouse prefrontal cortex (PFC) samples. Samples were collected on day 74 after footshock (“shock”) or mock treatment (“no-shock”) of male C57/BL6/N mice. Each row represents a miRNA and each column represents a sample. Dendrograms represent complete-linkage clustering (by Euclidean distance) between samples. The sample clustering tree is shown on the top. The color scale illustrates the intensities of the relative miRNA expression levels: decreased scores are represented in red and increased in green, with intensity encoding magnitude. Significant alterations in miRNA expression levels determined by pairwise *t*-tests are highlighted in bright pink (corr. *p* < 0.05), statistical trends (corr. *p* < 0.1) in bright blue. Note that two samples of the no-shock-fluoxetine group were excluded during data processing (shock-vehicle: *n* = 6 per group; shock-fluoxetine: *n* = 6; no-shock-fluoxetine: *n* = 4). Statistical procedures are explained in the Section “Materials and Methods” and data are presented in the Section “Results” Vehicle, drinking water (solvent of fluoxetine).

**Figure 4:**
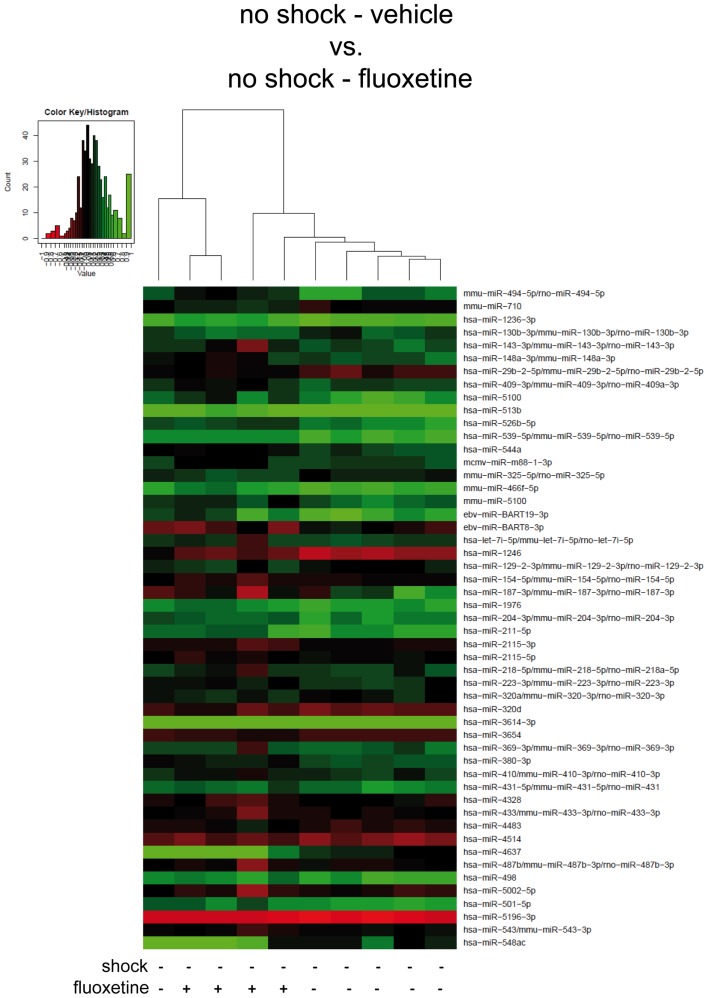
**In non-shocked mice, fluoxetine treatment does not significantly alter mouse PFC miRNA profiles in the long-term**. Here, results of the hierarchical cluster analysis (HCA) of the non-shocked vehicle-treated (no-shock-vehicle) versus the non-shocked fluoxetine-treated (no-shock-fluoxetine) groups are presented in a heatmap. The top 50 miRNA candidates with the lowest corrected *p*-values (resulting from pairwise comparison of the two groups shown here) were included. MiRNA expression levels were determined with LNA™ miRNA microarray analysis of mouse prefrontal cortex (PFC) total RNA samples. Samples were collected on day 74 after footshock (“shock”) or mock treatment (“no-shock”) of male C57/BL6/N mice (no-shock-vehicle: *n* = 6 per group: no-shock-fluoxetine: *n* = 4). Each row represents a miRNA and each column represents a sample. Dendrograms represent complete-linkage clustering (by Euclidean distance) between samples. The sample clustering tree is shown on the top. The color scale illustrates the intensities of the relative miRNA expression levels: decreased scores are represented in red and increased in green, with intensity encoding magnitude. See Section “Materials and Methods” for statistical procedures. Vehicle, drinking water (solvent of fluoxetine).

To avoid molecular analyses to be influenced by acute effects of the behavioral testing procedure, we harvested mouse brains 2 weeks after behavioral analyses. For preparation of total RNA and subsequent miRNA profile analyses, PFCs were dissected from six mice per group. With the aim to identify miRNA candidates regulated by traumatic stress and/or by fluoxetine treatment, we subjected all of these 24 PFC total RNA samples to miRNA microarray analysis. After background correction and normalization, expression values were subjected to pairwise *t*-testing (no-shock-vehicle versus shock-vehicle; shock-vehicle versus shock-fluoxetine; no-shock-fluoxetine versus shock-fluoxetine; no-shock-vehicle versus no-shock-fluoxetine) and the resulting *p*-values were Benjamini–Hochberg corrected. Then, miRNAs were ranked by corrected *p*-values and the resulting top 50 candidates, i.e., the miRNAs with the lowest *p*-values, were subjected to unsupervised HCA. We performed four HCAs in total (Figures 2–4).

First, we looked for miRNAs regulated by traumatic footshock: unsupervised HCA of footshocked versus non-shocked groups (both vehicle-treated) revealed that samples clustered, with one exception, according to treatment by their miRNA expression values (Figure 2). However, pairwise comparison of miRNA expression profiles showed that no miRNA was significantly differentially expressed between these two groups. Thus, traumatic footshock causes a long-lasting PTSD-like syndrome in mice (Figure 1) but does not significantly alter long-term miRNA expression in mouse PFC (Figure 2).

### Fluoxetine treatment significantly alters the expression of several miRNAs in the PFC of shocked mice

Then, we looked for the influence of fluoxetine treatment on miRNA profiles of shocked mice: unsupervised HCA of shocked fluoxetine-treated (shock-fluoxetine) versus shocked vehicle-treated (shock-vehicle) groups revealed that samples clustered perfectly according to treatment (Figure 3A). Moreover, comparison of these two groups, i.e., the shock-vehicle versus the shock-fluoxetine group, revealed, that therapeutic (Figure 1) fluoxetine treatment significantly reduced the relative expression of two miRNA candidates analyzed, namely of rno-miR-3559-3p [fold change (FC) 0.29, corrected *p* (corr. *p*) < 0.003] and of mmu-miR-1971 (FC 0.82, corr. *p* < 0.05) (Figure 3A, highlighted in bright pink) and furthermore decreased the expression of two other miRNAs [at least on the level of a trend toward statistical significance (i.e., with *p* < 0.1)], namely the expression levels of ebv-miR-BART8-3p (FC 0.53, corr. *p* < 0.06) and of mmu-miR-1947-3p (FC 0.67, corr. *p* < 0.06) (Figure 3A, highlighted in bright blue).

Next, to further dissect the individual contributions of traumatic stress and fluoxetine treatment on mouse PFC miRNA signatures, we compared the shock-fluoxetine group to the no-shock-fluoxetine group (Figure 3B) and to the no-shock-vehicle group (Figure 4). Two samples of the no-shock-fluoxetine group had to be excluded from microarray analysis during array data processing. HCA of the no-shock-fluoxetine group versus the shock-fluoxetine group showed that the two groups did not cluster correctly according to treatment. However, pairwise comparison of these two groups revealed that, the relative expression of mmu-miR-33-5p was enhanced, at least with a statistical trend (FC 1.26, corr. *p* < 0.07) and the relative expression of rno-miR-3559-3p was decreased, also with a statistical trend (FC 0.80, corr. *p* < 0.07) in PFC of shock-fluoxetine mice (Figure 3B, both highlighted in bright blue). Although the HCA of the no-shock-fluoxetine group versus the no-shock-vehicle group illustrates that, with one exception, the respective samples clustered according to treatment, pairwise comparisons of these two groups revealed no significant differences in miRNA profiles (Figure 4). Notably, mmu-miR-3559-3p emerged as a regulated miRNA candidate in two different pairwise comparisons since its expression was altered in shock-fluoxetine mice in comparison to both shock-vehicle (Figure 3A) and no-shock-fluoxetine mice (Figure 3B).

Taken together, microarray analyses revealed that, in shocked mice, on day 74 after subjection of mice to footshock, the therapeutic effect of fluoxetine (Figure 1) went along with a significant decrease in prefrontal cortical rno-miR-3559-3p and mmu-miR-1971 expression as well as with a trend of reduction in prefrontal cortical ebv-miR-BART8-3p and mmu-miR-1947-3p expression (Figure 3A). Finally, our analyses revealed that none of the miRNA candidates tested was altered by traumatic stress *per se* (Figure 2) or by fluoxetine treatment *per se* (Figure 4) which suggests that fluoxetine treatment interacts with traumatic stress to alter the expression levels of the mentioned miRNA candidates.

### RT-qPCR analysis confirmed that fluoxetine treatment alters the expression of mmu-miR-1971 and mmu-miR-33-5p in the PFC of shocked mice

**Figure 5:**
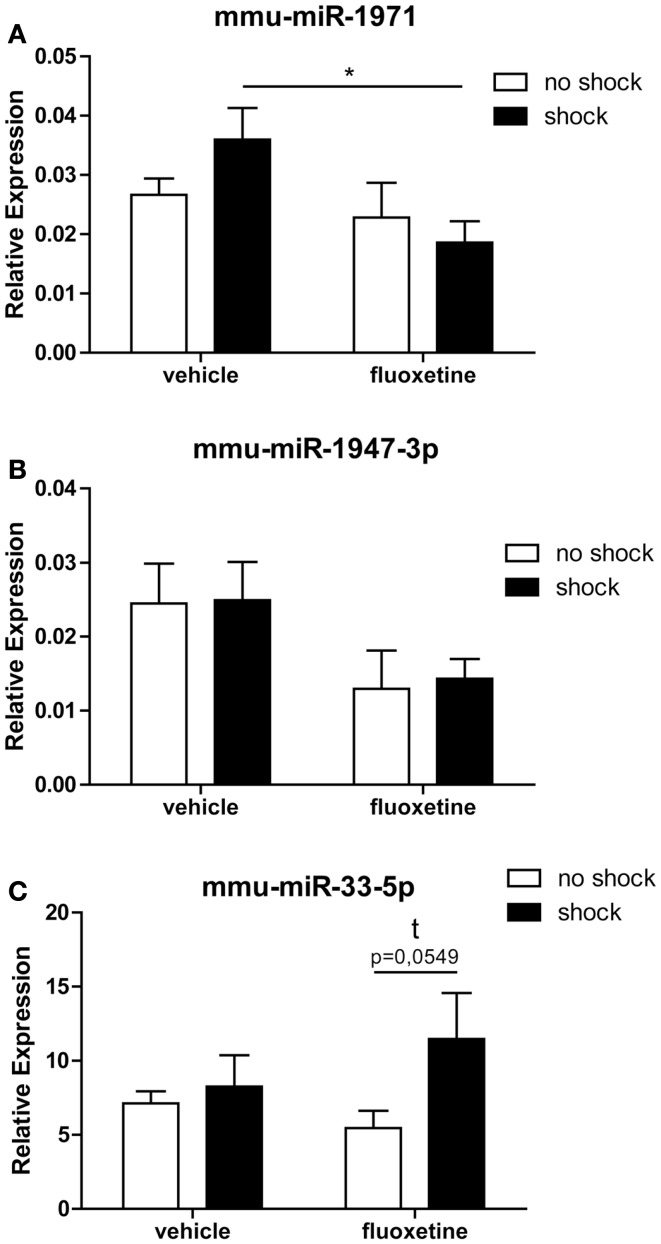
**RT-qPCR analysis confirmed that fluoxetine treatment alters the expression of mmu-miR-1971 and mmu-miR-33-5 in the PFC of shocked mice**. Depicted are results of the RT-qPCR analysis of the relative expression levels of the candidate microRNAs mmu-miR-1971 **(A)**, mmu-miR-1947-3p **(B)**, and mmu-miR-33-5p **(C)** compared between the no-shock-vehicle, no-shock-fluoxetine, shock-vehicle, and shock-fluoxetine groups (*n* = 6 per group). Prefrontal cortex (PFC) samples employed for RT-qPCR analyses were identical to those used for microarray analyses. Mmu-miR-100-5p was used for normalization using the ΔΔ*C*_t_ method. Presented data are means ± SEM. Statistical analysis was performed using two-way ANOVA and Bonferroni *post hoc* tests. Statistical significance of Bonferroni *post hoc* tests is indicated by *p < 0.05. See Section “Results” for statistical data. Vehicle, drinking water (solvent of fluoxetine).

Two out of the five array-identified miRNA candidates (rno-miR-3559-p, mmu-miR-1971, ebv-miR-BART8-3p, mmu-miR-1947-3p, mmu-miR-33-5p) could be validated by miRCURY LNA™ RT-qPCR: calculation of the statistical significance of RT-qPCR results with two-way ANOVA followed by Bonferroni *post hoc* correction confirmed a statistical trend toward a fluoxetine-mediated increase in prefrontal cortical mmu-miR-33-5p expression in shock-fluoxetine mice comparison to no-shock-fluoxetine mice (Figure 5C: Bonferroni posttest of shock-fluoxetine versus no-shock-fluoxetine: *t* = 2.205, *p* = 0.055). Most important, we observed a statistically significant reduction in mmu-miR-1971 expression in the PFC of shock-fluoxetine mice in comparison to shock-vehicle mice (Figure 5A: Bonferroni posttest of shock-vehicle versus shock-fluoxetine: *t* = 2.509, *p* < 0.050). RT-qPCR data do not allow the conclusion that fluoxetine *rescues* the footshock-induced increase in mmu-miR-1971 expression, since the latter failed to survive statistical correction (Figure 5A). Furthermore, despite a significant treatment effect in the two-way ANOVA (*F*_1,20 shock_ = 4.494, *p* = 0.030), Bonferroni posttests did not detect any significant fluoxetine-mediated changes in relative expression of mmu-miR-1947-3p. Thus, we cannot consider mmu-miR-1947-3p as a fully validated candidate (Figure 5B). Finally, despite repetitive tries and employment of optimized LNA™ -technology based miRNA primer sets, expression of rno-miR-3559-3p and ebv-miR-BART8-3p could not be detected by RT-qPCR. Given that, we speculate that the array-detected rno-miR-3559-3p and ebv-miR-BART8-3p signals might possibly represent technical artifacts.

In summary, the most important conclusion of this study is that in the PTSD mouse model studied here, the therapeutic action of fluoxetine (Figure 1) is accompanied by a significant reduction in prefrontal cortical mmu-miR-1971 expression on day 74 after shock exposure (Figures 3A and 5B).

### miRNA target prediction and gene ontology analysis

Finally, to get an idea of the potential role of mmu-miR-1971 and mmu-miR-33-5p in PTSD and of their general function, we performed an *in silico* analysis of target genes regulated by these two miRNA candidates: analysis performed with the miRWalk database^3^ (52) revealed several validated target genes of mmu-miR-33-5p (Table 3), but none of mmu-miR-1971. Then, we used computational methods to predict potential target genes of mmu-miR-1971: we applied TargetScanMouse 6.2^4^ (53) and MirTarget2^5^ (54, 55); we included only those predicted target genes that were identified with both approaches into subsequent GO analysis by employing GenericGeneOntologyTermFinder^6^ (56) and REViGO^7^ (57) (Table 2). The molecular functions of predicted mmu-miR-1971 target genes are mainly associated with small molecule and nucleic acid binding (Table 2). Moreover, most of them are involved in metabolic processes such as organic compound and RNA metabolism (Table 2).

**Table 2:** **Gene ontology analysis of predicted mmu-miR-1971 target genes**.

GO ID	GO term	Corr. *p*-value
**GO MOLECULAR FUNCTION**		
GO:0005488	Binding	8.98E−09
GO:0097159	Organic cyclic compound binding	2.97E−10
GO:0003723	RNA binding	6.98E−04
GO:0003676	Nucleic acid binding	2.54E−10
GO:0000166	Nucleotide binding	2.63E−04
GO:0036094	Small molecule binding	7.58E−04
GO:0043167	Ion binding	3.22E−05
GO:0005515	Protein binding	7.20E−05
GO:0003677	DNA binding	3.85E−05
**GO BIOLOGICAL PROCESS**		
GO:0006725	Cellular aromatic compound metabolic process	2.71E−06
GO:0008152	Metabolic process	2.50E−05
GO:0009987	Cellular process	1.02E−06
GO:0065007	Biological regulation	2.47E−06
GO:0006606	Protein import into nucleus	6.,19E−03
GO:0034654	Nucleobase-containing compound biosynthetic process	1.02E−05
GO:0046483	Heterocycle metabolic process	2.27E−06
GO:0044238	Primary metabolic process	6.15E−06
GO:0019438	Aromatic compound biosynthetic process	1.55E−05
GO:0071704	Organic substance metabolic process	3.73E−05
GO:0016071	mRNA metabolic process	7.31E−04
GO:0018130	Heterocycle biosynthetic process	1.65E−05
GO:0009058	Biosynthetic process	9.77E−05
GO:0000398	Nuclear mRNA splicing, via spliceosome	7.18E−04
GO:0044271	Cellular nitrogen compound biosynthetic process	2.77E−05
GO:0006807	Nitrogen compound metabolic process	8.77E−06
GO:0010468	Regulation of gene expression	4.09E−08
GO:0050794	Regulation of cellular process	1.33E−04
GO:0043170	Macromolecule metabolic process	1.36E−05
GO:0010467	Gene expression	5.25E−08
GO:0019222	Regulation of metabolic process	1.60E−04
GO:0044237	Cellular metabolic process	8.81E−06
GO:0016070	RNA metabolic process	8.04E−08
GO:0044260	Cellular macromolecule metabolic process	4.73E−06

*Indicated are gene ontology IDs (GO ID), gene ontology terms (GO term), and corrected p-values as determined by GenericGeneOntologyTermFinder (http://go.princeton.edu/cgi-bin/GOTermFinder) and REViGO (http://revigo.irb.hr/)*.

Then, to characterize possible molecular functions of validated mmu-miR-33-5p regulated targets, we performed GO analysis as described above. We found that most of the validated mmu-miR-33-5p targets are associated with protein, miRNA, and organic compound binding (Table 3). Furthermore, many of the validated mmu-miR-33-5p target genes are involved in cellular and developmental processes, and most important, in epigenetic regulation of gene expression and lipid metabolic processes (Table 3).

**Table 3:** **Gene ontology analysis of validated mmu-miR-33-5p target genes**.

GO ID	GO term	Annotated genes	Corr. *p*-value
**GO MOLECULAR FUNCTION**			
GO:0005488	Binding	Zp3, Lin28, Hprt1, Mos, H2afx, Ctdspl, H2afz, Fas, Rfpl4, Mt1, Ccnb2, Mbp, Dppa3, H1foo, Cd320, Dicer1, Hnt, Cpeb1, Srebf2, Ldlr, Cpt1a, Bmp4, Camk2g, Fgf21, Ccne1, Dnmt3b, Sycp3, Sirt6, Pou5f1, Abcg1	1.02E−06
GO:0035198	miRNA binding	Dicer1, Lin28, Pou5f1	8.49E−06
GO:0097159	Organic cyclic compound binding	H1foo, Cd320, Dicer1, Cpeb1, Srebf2, Lin28, Hprt1, Camk2g, Mos, H2afx, H2afz, Dnmt3b, Sycp3, Sirt6, Pou5f1, Abcg1	5.09E−03
GO:0005515	Protein binding	Zp3, Lin28, Hprt1, H2afx, H2afz, Fas, Rfpl4, Ccnb2, Mbp, Dppa3, Cd320, Dicer1, Cpeb1, Srebf2, Ldlr, Cpt1a, Bmp4, Camk2g, Fgf21, Ccne1, Dnmt3b, Sycp3, Abcg1, Pou5f1	1.02E−06
**GO BIOLOGICAL PROCESS**			
GO:0000003	Reproduction	H1foo, Dicer1, Zp3, Cpeb1, Lin28, Bmp4, Mos, H2afx, Ifitm3, Sycp3	5.11E−04
GO:0048610	Cellular process involved in reproduction	H1foo, Zp3, Cpeb1, Lin28, Sycp3, Bmp4, Mos, H2afx	4.52E−05
GO:0032502	Developmental process	Zp3, Lin28, Hprt1, H2afz, Fas, Ccnb2, Mbp, Dppa3, Hnt, Dicer1, Bmp4, Camk2g, Ccne1, Dnmt3b, Sycp3, Abcg1, Pou5f1	3.72E−03
GO:0042221	Response to chemical stimulus	Mbp, Dicer1, Srebf2, Lin28, Hprt1, Bmp4, Fgf21, Ifitm3, Fas, Dnmt3b, Mt1, Abcg1, Pou5f1	2.83E−03
GO:0071840	Cellular component organization or biogenesis	Hprt1, H2afx, H2afz, Fas, Ccnb2, Mbp, H1foo, Dppa3, Hnt, Dicer1, Cpeb1, Cpt1a, Bmp4, Dnmt3b, Sycp3, Abcg1, Pou5f1, Sirt6	1.33E−04
GO:0003133	Endodermal-mesodermal cell signaling	Bmp4, Pou5f1	3.54E−03
GO:0006325	Chromatin organization	Dppa3, H1foo, H2afx, H2afz, Sycp3, Dnmt3b, Pou5f1, Sirt6	1.36E−04
GO:0006259	DNA metabolic process	Dppa3, H1foo, Bmp4, H2afx, Ccne1, H2afz, Sycp3, Dnmt3b, Sirt6	2.32E−04
GO:0022402	Cell cycle process	H1foo, Dicer1, Cpeb1, Camk2g, Bmp4, Mos, H2afx, Sycp3, Ccnb2	5.36E−04
GO:0045595	Regulation of cell differentiation	Mbp, Dicer1, Hnt, Lin28, Bmp4, Ccne1, Fas, Dnmt3b, Pou5f1, Abcg1	6.50E−04
GO:0007049	Cell cycle	H1foo, Dicer1, Cpeb1, Camk2g, Bmp4, Mos, H2afx, Ccne1, Sycp3, Ccnb2	1.05E−03
GO:0006807	Nitrogen compound metabolic process	Zp3, Lin28, Hprt1, H2afx, H2afz, H1foo, Dppa3, Dicer1, Srebf2, Cpeb1, Cpt1a, Ldlr, Bmp4, Ccne1, Dnmt3b, Sycp3, Abcg1, Pou5f1, Sirt6	1.44E−03
GO:0040029	Regulation of gene expression, epigenetic	Dppa3, Dicer1, Dnmt3b, Lin28, Pou5f1	1.29E−03
GO:0048519	Negative regulation of biological process	Mbp, Dppa3, Dicer1, Zp3, Hnt, Srebf2, Lin28, Bmp4, Ifitm3, Fas, Dnmt3b, Mt1, Sycp3, Pou5f1, Abcg1	1.78E−03
GO:0045834	Positive regulation of lipid metabolic process	Zp3, Ldlr, Cpt1a, Abcg1	7.65E−03
GO:0016458	Gene silencing	Dicer1, Dnmt3b, Lin28, Pou5f1	7.65E−03
GO:0010033	Response to organic substance	Dicer1, Srebf2, Lin28, Hprt1, Bmp4, Fgf21, Ifitm3, Fas, Dnmt3b, Abcg1, Pou5f1	4.09E−03
GO:0050794	Regulation of cellular process	Zp3, Lin28, Hprt1, Mos, Ifitm3, Fas, Mt1, Ccnb2, Mbp, Dppa3, Hnt, Dicer1, Cpeb1, Srebf2, Ldlr, Cpt1a, Bmp4, Fgf21, Ccne1, Dnmt3b, Sycp3, Sirt6, Abcg1, Pou5f1	4.26E−03
GO:0071824	Protein-DNA complex subunit organization	H1foo, H2afz, Sycp3, H2afx	5.38E−03
GO:0080090	Regulation of primary metabolic process	Dppa3, Dicer1, Zp3, Cpeb1, Srebf2, Ldlr, Cpt1a, Lin28, Hprt1, Bmp4, Ccne1, Dnmt3b, Sirt6, Pou5f1, Ccnb2, Abcg1	7.43E−03
GO:0006323	DNA packaging	H1foo, H2afz, Sycp3, H2afx	7.37E−03
GO:0003130	BMP signaling pathway involved in heart induction	Bmp4, Pou5f1	3.54E−03
GO:0034641	Cellular nitrogen compound metabolic process	Zp3, Lin28, Hprt1, H2afx, H2afz, H1foo, Dppa3, Dicer1, Srebf2, Cpeb1, Cpt1a, Ldlr, Bmp4, Ccne1, Dnmt3b, Sycp3, Abcg1, Pou5f1, Sirt6	5.92E−04
GO:0050793	Regulation of developmental process	Mbp, Dicer1, Zp3, Hnt, Lin28, Bmp4, Ccne1, Fas, Dnmt3b, Pou5f1, Abcg1	1.71E−03
GO:0048523	Negative regulation of cellular process	Mbp, Dppa3, Dicer1, Zp3, Hnt, Srebf2, Lin28, Bmp4, Ifitm3, Fas, Dnmt3b, Mt1, Sycp3, Pou5f1, Abcg1	4.54E−04

*Indicated are gene ontology IDs (GO ID), gene ontology terms (GO term), the annotated gene names, and corrected p-values as determined by GenericGeneOntologyTermFinder (http://go.princeton.edu/cgi-bin/GOTermFinder) and REViGO (http://revigo.irb.hr/)*.

Taken together, target prediction and GO analyses revealed several predicted target genes of mmu-miR-1971 and several validated target genes mmu-miR-33-5p which might possibly be involved *inter alia* in PTSD pathobiology or fluoxetine-mediated alterations of molecular pathways. Interestingly, amongst these target genes we found none which had previously been repetitively associated with PTSD, like for instance FKBP5 (8), CDK5 (58), or synapsin (28).

## Discussion

Here, we present the first study exploring miRNA expression profiles in a PTSD mouse model. In summary, we demonstrate that the therapeutic action of fluoxetine in shocked mice (Figure 1) is correlated with a significant reduction in prefrontal cortical mmu-miR-1971 expression levels on day 74 after shock exposure (Figures 1 and 5A). The significance of this finding is supported by results of the unsupervised HCA of the shock-vehicle versus the shock-fluoxetine group which revealed that samples of both groups clustered perfectly according to treatment (Figure 3A) thereby demonstrating that the miRNome is a factor that contributes to biological differences in these two groups. RT-qPCR data do not allow the conclusion that fluoxetine *rescues* the footshock-induced increase in mmu-miR-1971 expression, since the latter failed to survive correction for multiple testing (Figure 5A). Furthermore, our analyses revealed a trend toward an increase of prefrontal cortical mmu-miR-33-5p expression in shock-fluoxetine mice in comparison to no-shock-fluoxetine mice (Figure 5C). Interestingly, we found that traumatic stress *per se* and fluoxetine treatment *per se* did not lead to significant alterations of mouse miRNA profiles on day 74 after trauma exposure (Figures 2 and 4) which suggests that fluoxetine interacts with traumatic stress to alter expression levels of mmu-miR-1971 and mmu-miR-33-5p (Figures 5A,C). To the best of our knowledge, these two miRNA candidates have not been associated with psychiatric disorders so far. MiR-1971 has hitherto not even been associated with the central nervous system (CNS). Instead, in the only study reporting expression level changes of miR-1971 demonstrated that, in the bone marrow, miR-1971 was differentially expressed between patients suffering from acute lymphoblastic leukemia (ALL) and healthy donors (59). However, to our knowledge, the in that study newly identified miRNA sequence, which was termed hsa-miR-1971 thereby representing it as the human homolog of murine mmu-miR-1971, is not annotated in miRBase 19.0. Our miRNA target prediction and GO analysis revealed that for miR-1971 no target genes have been validated so far (Table 2). Hence, regulation and function of miR-1971 are largely unexplored yet and await further studies. However, GO analysis of predicted miR-1971 target genes allude that this above-average small (18nt) miRNA candidate might be involved *inter alia* in basic metabolic processes like heterocycle and organic substance metabolism (Table 2: *p* = 2.27E−06 and *p* = 3.73E−05, respectively); neurotransmitters like serotonin or modulators of the serotonergic tone might belong to the organic substances whose metabolism is targeted by miR-1971, but, however, our GO analysis provided no direct hint for this speculation.

In contrast, miR-33 has been studied more intensely. A fundamental biological role of miR-33-5p (previous miRBase ID: miR-33) is suggested by the fact that, according to miRBase 18.0, its sequence is highly conserved in human, mouse, and rat. MiR-33 was found to be downregulated in the hippocampus of rats with status epilepticus (60), to regulate the cell cycle (61, 62), to be associated with mouse atherosclerosis (63, 64), as well as with metabolism of cholesterol (65, 66). The latter finding is supported by our GO analysis which revealed the biological process termed “positive regulation of lipid metabolic processes” to be significantly enriched among validated mmu-miR-33-5p target genes (Table 3: *p* = 7.65E−03). Low blood levels of cholesterol were found to be associated with suicidality (67, 68), while PTSD patients were repeatedly reported to exhibit elevated cholesterol blood levels (69, 70). Cholesterol biosynthesis in glial cells was shown to be influenced by fluoxetine and other antidepressants (59). In turn, most interestingly, there is strong evidence for an influence of the cholesterol metabolism on fluoxetine treatment response both in rodents (60) and in humans (61). The synopsis of these findings fuels the speculation that in the PTSD mouse model studied here, the shock × fluoxetine interaction-mediated increase of prefrontal cortical mmu-miR-33-5p expression (Figures 3B and 5C) might contribute to the previously reported influence of cholesterol metabolism on the response to fluoxetine treatment (59, 60). This synoptic speculation is worth addressing in future studies. However, even though our analyses do support an involvement of mmu-miR-33-5p in fluoxetine-mediated processes, they do neither speak for nor against the involvement of mmu-miR-33-5p in the *therapeutic* action of fluoxetine since we did *not* find significant mmu-miR-33-5p expression level differences between vehicle-treated and fluoxetine-treated shocked mice. Instead, our data suggest an involvement of mmu-miR-1971 in the therapeutic action of fluoxetine in footshocked mice.

Interestingly, fluoxetine was shown previously to regulate miRNA expression, namely to alter the expression of miR-16 in serotonergic raphe nuclei (71). However, in the here presented study which analyzed miRNA expression levels in another brain region, i.e., the PFC, in a different experimental context, miR-16 expression was not significantly altered by fluoxetine treatment. Since changes in miRNA expression can occur rapidly (72), we suppose that both traumatic stress and fluoxetine treatment might exert even stronger effects on miRNA expression at earlier time-points after challenge. At the late posttrauma time-point tested here, the *consequences* of trauma-stress and fluoxetine-mediated alterations of miRNA expression probably dominate alterations in miRNA expression itself. Moreover, it would also be interesting to evaluate miRNA profiles in other brain regions associated with PTSD, like for instance the hippocampus and the amygdala (31). To check for the generalizability of the results presented here, our findings should be validated in another mouse cohort and in other animal models for PTSD. Most important, it also remains to be tested, for instance by employing an *in vivo* knockdown approach, whether the miRNA candidates identified here *causally* contribute to the therapeutic effects of fluoxetine or not.

All in all, this study is the first that examined miRNA profiles in connection with PTSD and represents a promising starting point for further evaluation of the role of miRNAs in PTSD pathobiology.

## Conflict of Interest Statement

Florian Holsboer reports to be co-inventor of the patent “Genes associated with post-traumatic stress disorder (PTSD)”, international application number: PCT/EP2009/061890. All the other authors declare no conflict of interest since the Horst Kübler foundation, Bad Ragaz, Switzerland, which partly sponsored this study (consumables) had no role in study design, data collection and analysis, or preparation of the manuscript or decision to publish.

## Footnotes

^1^www.mirbase.org

^2^http://www.hiv.lanl.gov/content/sequence/HEATMAP/heatmap.html

^3^http://www.umm.uni-heidelberg.de/apps/zmf/mirwalk/mirnatargetpub.html

^4^http://www.targetscan.org/mmu_61/

^5^http://mirdb.org/miRDB/

^6^http://go.princeton.edu/cgi-bin/GOTermFinder

^7^http://revigo.irb.hr/
